# CAM-Vtrans: real-time sports training utilizing multi-modal robot data

**DOI:** 10.3389/fnbot.2024.1453571

**Published:** 2024-10-11

**Authors:** Hong LinLin, Lee Sangheang, Song GuanTing

**Affiliations:** ^1^College of Physical Education, Jeonju University, Jeonju, Jeollabuk-do, Republic of Korea; ^2^Gongqing Institute of Science and Technology, Jiujiang, Jiangxi Province, China

**Keywords:** assistive robotics, human-machine interaction, balance control, movement recovery, vision-transformer, CLIP, cross-attention

## Abstract

**Introduction:**

Assistive robots and human-robot interaction have become integral parts of sports training. However, existing methods often fail to provide real-time and accurate feedback, and they often lack integration of comprehensive multi-modal data.

**Methods:**

To address these issues, we propose a groundbreaking and innovative approach: CAM-Vtrans—Cross-Attention Multi-modal Visual Transformer. By leveraging the strengths of state-of-the-art techniques such as Visual Transformers (ViT) and models like CLIP, along with cross-attention mechanisms, CAM-Vtrans harnesses the power of visual and textual information to provide athletes with highly accurate and timely feedback. Through the utilization of multi-modal robot data, CAM-Vtrans offers valuable assistance, enabling athletes to optimize their performance while minimizing potential injury risks. This novel approach represents a significant advancement in the field, offering an innovative solution to overcome the limitations of existing methods and enhance the precision and efficiency of sports training programs.

## 1 Introduction

In the field of sports technology, the application of deep learning and machine learning techniques to enhance training efficiency and athlete performance has become a hot topic of research (Zheng et al., 2020). These technologies can accurately analyze athletes' movements and provide real-time feedback, helping athletes improve their skills more effectively (Pan et al., 2019). However, while existing technologies can handle single data sources such as video or biosensor data, their capabilities are still insufficient when it comes to integrating and processing multiple types of data (Herman et al., 2021), especially when simultaneously dealing with visual information and verbal instructions. This limitation highlights the need for the development of new methods to comprehensively understand and guide athlete training.

Traditional methods primarily rely on symbolic AI and knowledge representation for Taekwondo action recognition. Expert systems, for example, simulate human experts' decision-making processes by encoding their knowledge and provide explicit explanations for each recognition result. Yang et al. (2021) proposed a multi-knowledge representation framework for big data AI applications. Additionally, a comprehensive review by Himabindu et al. (2023) showcased the combination of symbolic reasoning and deep learning in neural-symbolic AI, highlighting its various applications and developments across different domains. Rule-based methods, on the other hand, utilize a set of predefined rules for action recognition. These methods demonstrate high determinism and reliability, performing well even in the face of complex or diverse actions. Jin et al. (2022) introduced a deep reinforcement learning system for automatic symbol grounding discovery, while the research by Ilager et al. (2023) showcased the cost-saving benefits of symbolic representation in edge AI applications. Furthermore, logistic regression, as a statistical method, learns features from training data for classification decisions. It not only finds important applications in action recognition but also significantly improves classification accuracy. The study by Insuasti et al. (2023) demonstrated the application of logistic regression in sports action recognition, while Wu et al. (2022) further explored the use of fuzzy logic in symbolic representation, enhancing the symbolic foundations of AI. These methods offer advantages such as strong interpretability and transparency in the decision-making process. However, these methods have limitations in handling complex and diverse actions as well as limited capabilities in processing large-scale data.

To address the limitations of traditional algorithms, data-driven and machine learning-based approaches have been employed in multi-modal robot-assisted sports training. These approaches mainly utilize methods such as decision trees, random forests, and multi-layer perceptrons to tackle the challenges. This approach offers advantages such as efficient handling of large-scale data, high accuracy, and the ability to handle non-linear problems. For instance, Tjondronegoro and Chen (2006) automated event classification in sports videos using decision tree methods, while Jose et al. (2023) applied decision tree algorithms in predicting athlete performance. Furthermore, Morciano et al. (2023) used random forest algorithms to predict performance indicators of soccer players, demonstrating their superiority in handling biomechanical data, and Yagin et al. (2023) showcased the high accuracy of random forests in determining the positions of professional soccer players. Lastly, Aresta et al. (2022) highlighted the superior performance of multi-layer perceptrons in classifying elite and novice fencers based on biomechanical data, while Bakthavatchalam et al. (2022) demonstrated the efficient predictive performance of multi-layer perceptrons in agriculture. However, these methods have challenges such as overfitting, high computational costs, and strong reliance on large amounts of annotated data.

To overcome the limitations of statistical and machine learning algorithms, deep learning-based approaches have been used for Taekwondo action recognition, primarily employing Convolutional Neural Networks (CNN), reinforcement learning, and Transformer models. These methods offer higher accuracy and the ability to handle complex data. Firstly, Convolutional Neural Networks efficiently extract image features and have shown remarkable performance in predicting sports game outcomes and recognizing athlete actions. For example, Chen et al. (2020) used CNN to predict NBA game results with an accuracy of 91%, while Liu (2022) utilized CNN to improve action detection rates in sports videos. Secondly, reinforcement learning demonstrates significant potential in sports training by continuously adjusting strategies to optimize the decision-making process. The reinforcement learning approach proposed by Jia et al. (2020) improved players' winning rates in basketball training, and the research by Du et al. (2021) showcased the application of reinforcement learning in esports. Lastly, Transformer models, known for their advantages in handling sequential data, have been used for time-series analysis of motion signals, showing impressive performance. Dirgová Luptáková et al. (2022) achieved 99.2% accuracy in human activity recognition using the Transformer model, while Hauri and Vucetic (2023) combined Transformer with LSTM for team activity recognition in basketball games. However, these methods have challenges such as high computational complexity and a demand for large-scale training data.

Considering these challenges, this study proposes a novel approach, CAM-Vtrans: Real-time Sports Training Utilizing Multi-modal Robot Data, to address the limitations of traditional and machine learning algorithms, such as poor adaptability to complex environments, high computational costs, and dependency on large labeled datasets. CAM-Vtrans combines Vision Transformer (ViT), CLIP, and cross-attention mechanisms. ViT divides the image into multiple small patches and encodes them as a sequence, utilizing the self-attention mechanism to process these sequences and capture complex relationships within the image. This approach is particularly effective in handling sports activity images with rich details. The introduction of the CLIP model enables the system to understand training instructions in natural language and combines them with visual data to provide context-aware feedback. Through the cross-attention mechanism, this system further optimizes the fusion of different modalities, making the transformation from visual information to language descriptions more accurate and efficient. This integrated approach not only enhances the accuracy and efficiency of sports training analysis but also significantly reduces the computational burden and reliance on extensive labeled data.

The main contributions of this research can be summarized as follows:

- CAM-Vtrans is an innovative approach that combines Vision Transformer (ViT), the CLIP model, and cross-attention mechanisms to process and analyze multi-modal robot data in real-time, enhancing the accuracy of feedback in sports training.
- This method performs exceptionally well in various multi-scenario applications, efficiently handling complex sports activity images. It possesses broad applicability and adaptability, providing reliable support for different training requirements.
- Experimental results demonstrate that CAM-Vtrans significantly outperforms traditional methods in action recognition and feedback accuracy, greatly improving the effectiveness of sports training while reducing computational costs and reliance on large-scale annotated data.

## 2 Related work

### 2.1 Assisting sports training

In recent years, machine learning has made significant progress in assisting sports training tasks. Traditional sports training methods heavily rely on coaches' experience and intuition, which often suffer from subjectivity and lack of precision. The introduction of machine learning has made the training process more scientific and systematic. Classic machine learning algorithms such as decision trees, random forests, and logistic regression have been widely applied in areas such as athlete performance prediction and injury risk assessment. For example, decision tree-based systems can provide personalized training recommendations by analyzing athletes' physiological and training data (Jose et al., 2023). However, these traditional machine learning methods also have some notable drawbacks and limitations (Tang et al., 2023). Firstly, these methods require high-quality and large quantities of labeled data to train models, which can be costly to acquire. Moreover, traditional machine learning algorithms exhibit limitations when dealing with complex and multi-dimensional sports data. For instance, while random forests can handle non-linear relationships to some extent, they still struggle with highly complex and dynamically changing sports data (Morciano et al., 2023). Additionally, these methods lack interpretability and explainability, making it difficult to provide clear explanations for training outcomes and limiting their practical applications (Dong et al., 2024). To overcome these limitations, deep learning methods have gradually become a research focus in the field of sports training. Deep learning, by constructing multi-layer neural networks, can better capture complex features and patterns, thus improving the predictive accuracy and robustness of models. However, deep learning methods also face challenges such as high computational costs, long training times, and dependence on large-scale annotated data, which still need to be further addressed in practical applications (Wang et al., 2024)

### 2.2 Transformer models

Since its introduction in 2017, the Transformer model has achieved groundbreaking advancements across multiple domains. Its unique self-attention mechanism and parallel processing capabilities have made Transformers particularly prominent in natural language processing (NLP) tasks. For instance, models like BERT and GPT, which are based on Transformer architecture, have demonstrated significant effectiveness in tasks such as language understanding, text generation, and machine translation. The Transformer model addresses the inefficiencies and vanishing gradient problems associated with traditional sequential models like RNNs and LSTMs by processing input sequences in parallel and dividing them into smaller chunks (Lu et al., 2024). Beyond NLP, the Transformer model has also shown strong capabilities in the field of computer vision (CV). Vision Transformer (ViT), by dividing images into fixed-size patches and processing these patches as input sequences, has achieved performance comparable to or even surpassing that of convolutional neural networks (CNNs). ViT has excelled in tasks such as image classification, object detection, and image segmentation, proving the potential of Transformers in handling visual data (Hu et al., 2019). In addition, the Transformer model has wide-ranging applications in time series data analysis, recommendation systems, and game AI. In time series data analysis, Transformers can effectively capture long-term dependencies, enhancing prediction accuracy. In recommendation systems, Transformers model user behavior sequences to provide more precise recommendations. In game AI, Transformers, combined with deep reinforcement learning, optimize strategy selection.

### 2.3 Sports action recognition

Sports action recognition is a crucial research area in sports science and computer vision, aiming to automatically identify and evaluate athletic performance by analyzing athletes' motion data. Traditional action recognition methods primarily rely on feature engineering-based machine learning algorithms, such as support vector machines, decision trees, and random forests. These methods extract features from motion data for classification and recognition, achieving certain levels of effectiveness (Zhao et al., 2020). With the development of deep learning technologies, the advantages of convolutional neural networks (CNNs) in image and video processing have become increasingly apparent, leading to their widespread application in sports action recognition. CNNs can automatically learn and extract high-level features from data, significantly improving the accuracy and robustness of action recognition (Zou et al., 2019). Additionally, temporal models in deep learning, such as long short-term memory networks (LSTMs) and Transformer models, have been applied to action recognition, better handling time series data and capturing dynamic changes in actions. However, despite the impressive performance of deep learning methods in action recognition, several challenges and limitations persist. First, deep learning models require large-scale annotated data, and acquiring and annotating sports action data is costly, limiting the effectiveness of model training. Second, deep learning models have high computational complexity, requiring substantial computational resources and time for training and inference, which can be a bottleneck in real-time applications. Moreover, existing action recognition models still face difficulties in handling complex and diverse actions, making it challenging to adapt to various sports scenarios and action types. To address these issues, researchers are exploring multi-modal data fusion methods, combining visual, auditory, and tactile data to enhance the accuracy and robustness of action recognition (Li et al., 2018). Additionally, emerging technologies such as reinforcement learning and self-supervised learning are being introduced to action recognition to reduce reliance on annotated data and improve model generalization. Despite these advancements, achieving efficient, accurate, and robust sports action recognition remains a challenging research topic, necessitating further exploration and innovation.

## 3 Methodology

### 3.1 Overview of our network

In this research, we propose a multimodal robotic system that combines Vision Transformer (ViT), CLIP, and cross-attention mechanisms for real-time feedback and guidance in sports training. The main innovation of this system lies in the use of advanced visual and language processing models to analyze athletes' performances in-depth and provide immediate guidance and feedback.

**Figure 1:**
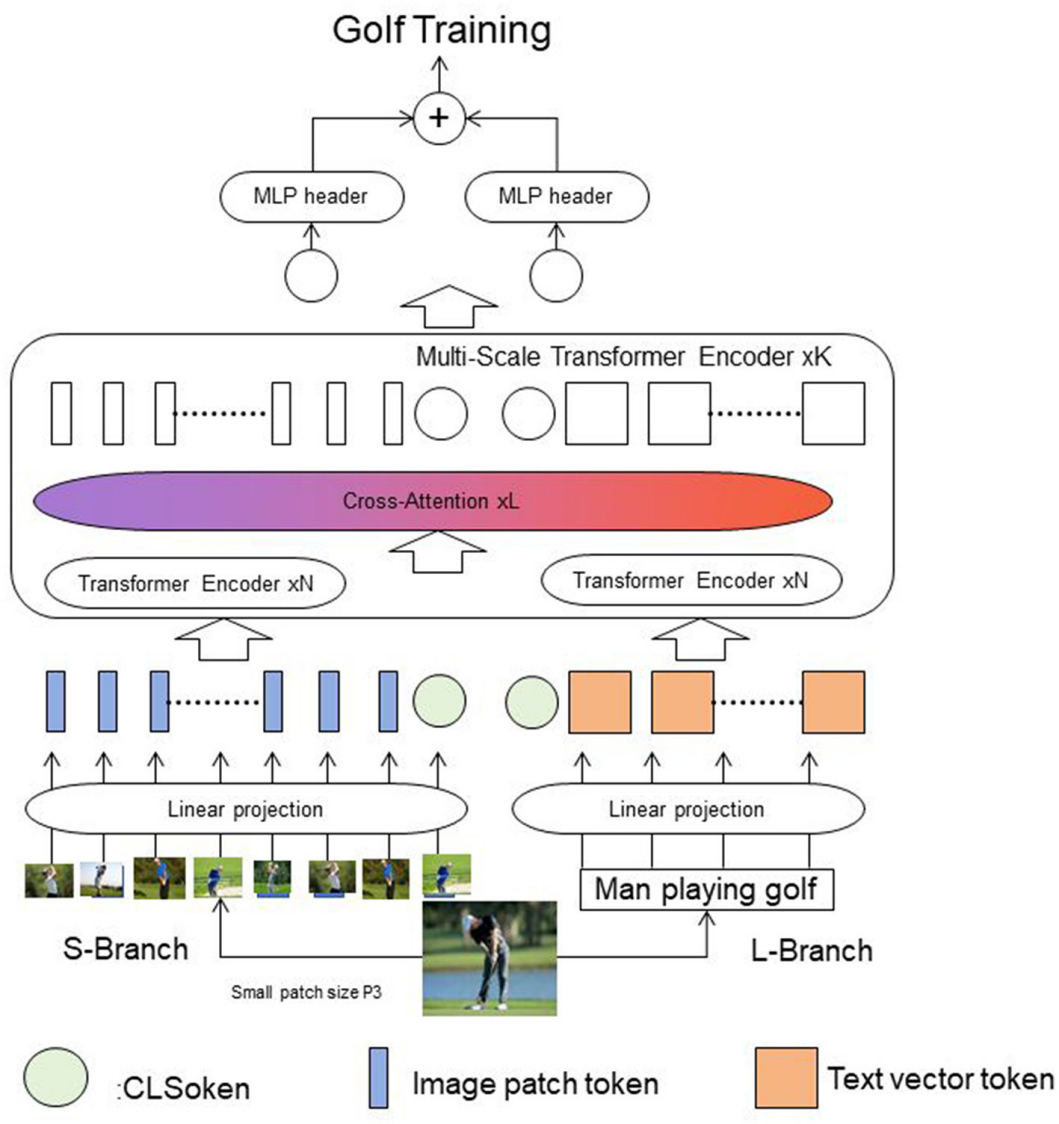
The overall framework diagram of the proposed method is presented.

Figure 1 shows the overall framework diagram of the proposed method.

Textual information is inputted from the L-Branch branch and is segmented into words or subwords. The text is then transformed into fixed-dimensional vector representations through an embedding layer. These text vectors are linearly projected and inputted into the corresponding Transformer Encoder. Images are inputted from the S-Branch, and each branch's image is divided into fixed-sized patches. After linear projection, the image patches are inputted into their respective Transformer Encoders. The image and text features interact and fuse through a Cross-Attention mechanism. The Cross-Attention layer takes features from the image and text encoders, calculates the correlation between them, and generates a fused multimodal feature representation. The fused multimodal features are further processed by a Multi-Scale Transformer Encoder layer to capture features at different scales, enhancing the expressive power of the features. Finally, a Multi-Layer Perceptron (MLP) head is used for tasks such as classification or regression. In the revised version, we will update Figure 1 to visually illustrate the processing and flow of textual information, including adding a schematic diagram of text input, demonstrating the processing of text through the embedding layer and linear projection layer, and clarifying the interaction between image and text features in the Section 3.4.

Differentiation from prior work: While the combination of ViT, CLIP, and Cross-Attention has been proposed in other domains, our research is the first to apply it to real-time sports coaching systems. Unlike previous studies, our research focuses on effectively integrating visual and textual data in dynamic and real-time sports training environments. Specifically, our proposed CAM-Vtrans system takes into account the continuity and complexity of sports actions during its design. Through optimized Cross-Attention mechanisms and multi-scale feature extraction modules, the system is able to provide stable and accurate feedback even with high-frequency inputs.

Overcoming limitations of previous methods: Previous methods often suffer from low computational efficiency and long feedback latency when dealing with real-time multimodal data. In this research, we address these limitations by introducing the ViT-Adapter module, which enhances feature extraction efficiency. Through optimized Cross-Attention mechanisms, we achieve faster and more accurate multimodal data fusion. Compared to traditional single-modal or inefficient multimodal methods, the CAM-Vtrans system significantly reduces inference time and improves the accuracy of real-time feedback, overcoming the limitations of previous methods in terms of real-time performance and data fusion.

Reasons for method selection: We chose the combination of ViT, CLIP, and Cross-Attention because these techniques have demonstrated excellent performance in handling complex visual and textual data. ViT is renowned for its powerful visual feature extraction capabilities, while CLIP effectively maps visual and textual data to the same feature space, enabling cross-modal understanding. The Cross-Attention mechanism efficiently establishes correlations between different modalities, enhancing information fusion. These characteristics make them well-suited for application in sports training scenarios that involve large amounts of visual and textual data and require real-time feedback. Therefore, the selection of these methods is not random but based on their superiority in multimodal data processing and real-time performance.

Firstly, the Vision Transformer (ViT) is employed to process video data captured from multiple cameras. ViT divides each frame into several image patches, converts these patches into a sequence of vectors, and processes them with self-attention mechanisms to identify key visual information. This approach allows the model to focus on specific regions within the image that are relevant to the movement technique, improving the accuracy and granularity of motion analysis. Simultaneously, the CLIP model is utilized to process and parse natural language inputs such as coach instructions or verbal feedback from athletes. CLIP learns from a large corpus of image-text pairs, establishing intuitive associations between image content and textual descriptions. This enables CLIP to directly relate language descriptions to visual data, providing robust support for precise understanding of movement techniques and coach's intentions. In the implementation workflow, once the athlete starts training, the system collects video and audio data in real-time. The visual and language data are processed separately by ViT and CLIP, respectively, and then fed into the cross-attention layer. In this layer, the system analyzes the correlations and interactions between visual and language information, optimizing the fusion process to extract the most valuable insights from the inputs. The core of the cross-attention mechanism lies in its ability to dynamically adjust the focus on different data sources based on specific training scenarios, providing more personalized and goal-oriented training recommendations. After performing these analyses, the system generates specific feedback reports, including action correction guidelines, performance evaluations, and improvement suggestions. This feedback can be presented directly to the athlete through a graphical user interface or sent to the coach via mobile devices. Additionally, the system includes a feedback adjustment module that allows the coach to fine-tune the level and frequency of feedback as needed, ensuring training continuity and adaptability. The focal point of the entire system design is to ensure real-time and accurate feedback, making the training process more intelligent and efficient. The aim is to maximize athletes' performance and training effectiveness through technological means.

### 3.2 Vision-transformer

**Figure 2:**
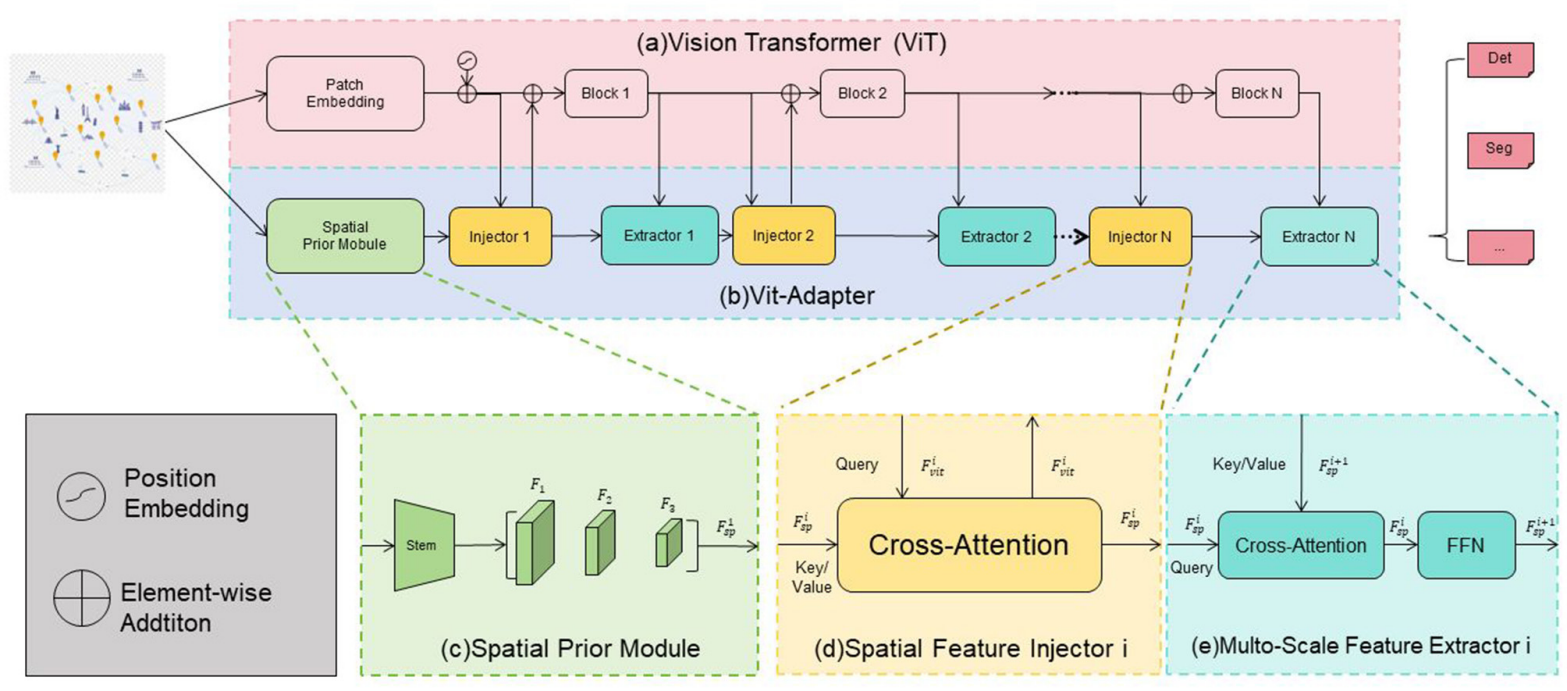
A schematic diagram of the principle of vision-transformer model.

Vision Transformer (ViT) (Miyazawa et al., 2022) is a deep learning model that applies the Transformer architecture to process visual data. Traditionally, Convolutional Neural Networks (CNNs) have been the dominant approach for visual tasks, but ViT introduces a novel paradigm by leveraging the self-attention mechanism of Transformers (Papadakis and Spyrou, 2024). Figure 2 is a schematic diagram of the principle of Vision-transformer Model.

The ViT-Adapter consists of the following components: Firstly, the Spatial Prior Module is responsible for initially extracting spatial features from the input image. The image first goes through the Stem layer, generating a series of feature maps (F1, F2, F3, …, Fsp) that capture spatial information at different scales, preparing for the subsequent feature injection. Secondly, the Spatial Feature Injector is one of the key modules of the ViT-Adapter. It injects the spatial features (Fsp) extracted by the Spatial Prior Module into the intermediate features (Fvit) of the ViT using a Cross-Attention mechanism. Specifically, the intermediate features of the ViT serve as the Query, while the spatial features act as the Key and Value. The Cross-Attention calculates the fused features (Fsp + Fvit). Then, the Multi-Scale Feature Extractor further processes the fused features through multiple Cross-Attention layers and a Feed-Forward Neural Network (FFN) to enhance the expressive power of multi-scale features, enabling the model to better capture image details and global information. Additionally, the ViT-Adapter inserts Injector and Extractor modules between each block of the ViT. The Injector module injects the features from the Spatial Prior Module into the current ViT features, while the Extractor module extracts useful information from the fused features for the next Transformer Block to use. Finally, after being processed by multiple Transformer Blocks and ViT-Adapter modules, the final features are fed into a Multi-Layer Perceptron (MLP) head for tasks such as classification, detection, or segmentation. Through these improvements, the ViT-Adapter significantly enhances the ViT model's ability to capture spatial features when processing images, improving its performance in various visual tasks.

The Vision Transformer (ViT) model operates by dividing an input image into smaller patches, which are then flattened into a sequence of 1D vectors capturing local visual information. These patches are linearly projected into higher-dimensional embeddings, serving as the input to the Transformer model. The Transformer architecture, composed of multiple identical layers each containing a self-attention mechanism and a feed-forward neural network, captures both global and local dependencies within the sequence of patches. During self-attention, patches exchange information and capture long-range dependencies, with attended representations aggregated and combined with original patch representations using residual connections. This process refines the patch representations based on contextual information. After multiple layers, the final image representation is obtained, which can be used for tasks like image classification, object detection, or segmentation. ViT's advantages include capturing global and local information, scalability, and learning from raw pixels without hand-engineered features. However, its self-attention mechanism's quadratic computational complexity is a limitation. In real-time feedback for multimodal robots in sports training, ViT analyzes visual information to understand and provide guidance on body movements. Trained on annotated sports videos, ViT extracts relevant features and captures spatial relationships, enabling the robot to offer accurate, context-aware feedback by leveraging self-attention to focus on critical image regions and dependencies between patches.

The input image is divided into patches, resulting in a sequence of patches, denoted by *x*_*i*_, where *i* represents the index of each patch. Each patch is then linearly projected to a higher-dimensional embedding space using a learnable linear transformation. Let's denote the projected embeddings as *z*_*i*_.

The self-attention mechanism in ViT is defined by the following equations (Equation 1):


(1) $$\begin{array}{l} \text{Attention}\left(Q,K,V\right)=\text{softmax}\left(\frac{QK^{T}}{\sqrt{d_{k}}}\right)V \end{array}$$

Here, *Q*, *K*, and *V* are the query, key, and value matrices, respectively. They are derived from the projected embeddings *z*_*i*_ as follows:


(2) $$\begin{array}{l} Q=ZW_{Q}\;K=ZW_{K}\;V=ZW_{V} \end{array}$$

In these equations, *Z* is the matrix obtained by stacking the projected embeddings *z*_*i*_, and *W*_*Q*_, *W*_*K*_, and *W*_*V*_ are learnable linear transformation matrices (Equation 2).

The self-attention mechanism calculates the attention weights between patches by computing the dot product similarity between the query and key matrices, scaled by the square root of the dimension *d*_*k*_. The softmax function is applied to obtain the attention weights, which are then used to weight the values *V*.

The attended representations are computed as follows (Equation 3):


(3) $$\begin{array}{l} \text{SelfAtt}\left(Z\right)=\text{Attention}\left(Q,K,V\right) \end{array}$$

The attended representations are then combined with the original patch embeddings using a residual connection, resulting in the intermediate representations:


(4) $$\begin{array}{l} \text{Intermediate}\left(Z\right)=\text{LayerNorm}\left(Z+\text{SelfAtt}\left(Z\right)\right) \end{array}$$

Here, LayerNorm denotes layer normalization (Equation 4).

The intermediate representations are then passed through a feed-forward neural network (FFN) with two linear transformations and a non-linear activation function, typically a GELU activation:


(5) $$\begin{array}{l} \text{FFN}\left(Z\right)=\text{GELU}\left(\text{Intermediate}\left(Z\right)W_{1}+b_{1}\right)W_{2}+b_{2} \end{array}$$

*W*_1_, *W*_2_, *b*_1_, and *b*_2_ are learnable parameters of the feed-forward network (Equation 5).

The output of the ViT model is obtained by stacking multiple layers of self-attention and feed-forward networks. The final representation of the image is typically obtained by applying mean pooling to the patch embeddings.

In summary, Vision Transformer is a powerful model for visual processing that replaces traditional convolutional approaches with self-attention mechanisms. Its ability to capture global and local dependencies makes it well-suited for understanding and analyzing visual data in real-time feedback and guidance systems for multimodal robots in sports training.

### 3.3 CLIP

**Figure 3:**
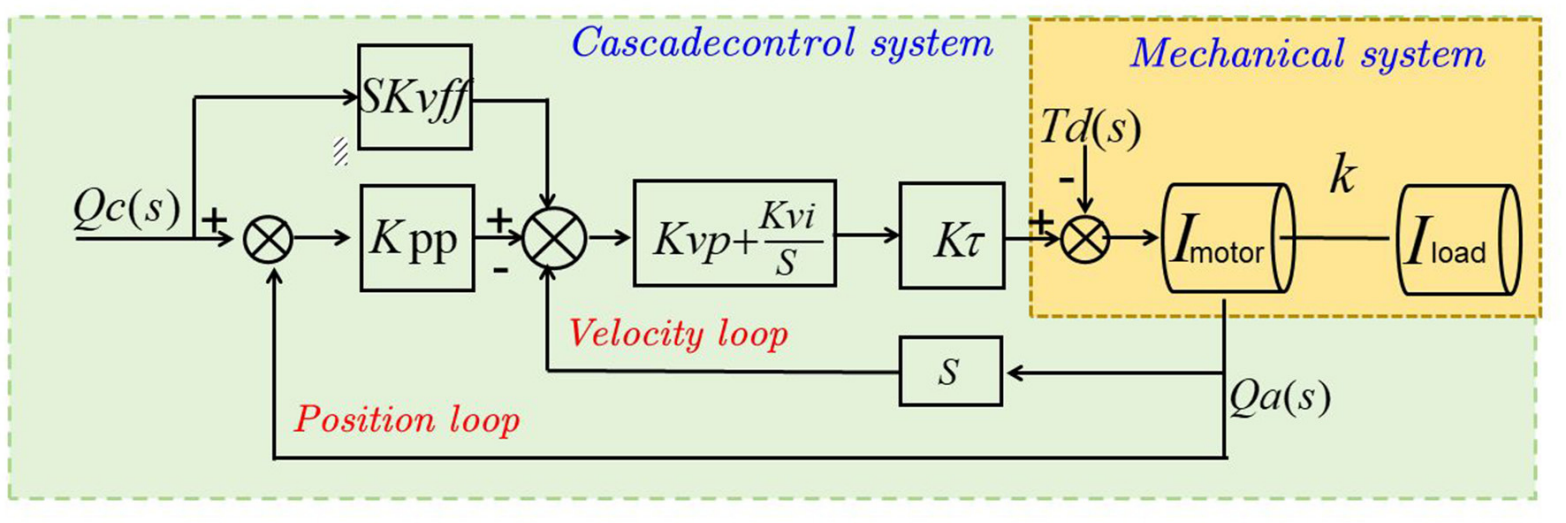
A schematic diagram of the principle of CLIP model.

CLIP (Contrastive Language-Image Pretraining) is a deep learning model that learns to associate images and their corresponding text descriptions (Dobrzycki et al., 2023). It aims to bridge the gap between vision and language modalities, enabling cross-modal understanding and reasoning. The key idea behind CLIP is to leverage large-scale pretraining on a dataset of image-text pairs, allowing the model to learn rich representations that capture the semantic relationship between visual and textual information (Koh et al., 2024). The basic principle of the CLIP model involves jointly training a vision encoder and a text encoder. The vision encoder processes images and maps them to a high-dimensional latent space, while the text encoder processes textual descriptions and maps them to the same latent space. The encoders are trained to ensure that corresponding image-text pairs are closer to each other in the latent space compared to non-corresponding pairs. Figure 3 is a schematic diagram of the principle of CLIP Model.

The training process of CLIP involves several key steps: first, the input image is encoded by a vision encoder, typically a convolutional neural network (CNN), which extracts visual features and projects them into a latent space using a learnable linear transformation. Simultaneously, the input text description is encoded by a text encoder based on a Transformer architecture, which tokenizes the text, applies word embeddings, and processes it through multiple Transformer layers to produce the text's representation in the latent space. CLIP utilizes a contrastive loss function to maximize the similarity between corresponding image-text pairs while minimizing the similarity between non-corresponding pairs, achieved by measuring the cosine similarity between their latent representations. Pretraining on large-scale datasets, such as Conceptual Captions and ImageNet, enables CLIP to learn generalizable representations capturing the semantic relationship between images and texts. After pretraining, CLIP can be fine-tuned for downstream tasks like image classification, object detection, or image captioning. In real-time feedback and guidance for multimodal robots in sports training, CLIP is crucial for understanding and associating visual and textual information. By aligning and reasoning about sports movements based on annotated image-text pairs, CLIP allows the robot to understand textual annotations related to key movements, techniques, and performance indicators. Leveraging the pretrained CLIP model, the robot can generate real-time feedback and guidance based on its comprehension of the athlete's movements and the semantic context provided by textual information.

Let's consider an image-text pair with an image *I* and a text description *T*.

Image Encoding: The image *I* is processed by a vision encoder, typically a convolutional neural network (CNN), to extract visual features. Let's denote the image representation as *v*_*I*_. Text Encoding: The text description *T* is processed by a text encoder, typically a Transformer-based architecture, to encode the textual information. Let's denote the text representation as *v*_*T*_. Similarity Measurement: The similarity between the image and text representations is measured using cosine similarity. It can be calculated as:


(6) $$\begin{array}{l} \text{Similarity}\left(v_{I},v_{T}\right)=\frac{v_{I}\cdot v_{T}}{\left|v_{I}|\cdot |v_{T}\right|} \end{array}$$

Here, · denotes the dot product, and |·| represents the Euclidean norm (Equation 6).

Contrastive Loss: CLIP utilizes a contrastive loss function to train the model. Given a positive pair (an image-text pair that matches) and a set of negative pairs (image-text pairs that do not match), the contrastive loss encourages the positive pair to have a higher similarity than the negative pairs. The contrastive loss can be formulated as:


(7) $$\begin{array}{l} \text{Loss}=-\log\left(\frac{\exp\left(\text{Similarity}\left(v_{I},v_{T}\right)\right)}{\overset{N}{\underset{j=1}{\sum }}\exp\left(\text{Similarity}\left(v_{I},v_{T_{j}}\right)\right)}\right) \end{array}$$

Here, *N* represents the number of negative pairs, and *v*_*T*_*j*__ denotes the text representation of the *j*-th negative pair (Equation 7).

The loss function aims to maximize the similarity between the positive image-text pair while minimizing the similarities between the positive pair and negative pairs. During training, the model optimizes the parameters of the image and text encoders to minimize the contrastive loss. This process enables the model to learn representations that associate images and their corresponding text descriptions. In summary, CLIP is a powerful model that combines image and text encoders to learn joint representations of visual and textual information. Its large-scale pretraining on image-text pairs enables it to capture the semantic relationship between these modalities. In the context of real-time feedback and guidance in sports training, CLIP enhances the multimodal robot's understanding and reasoning capabilities, facilitating personalized feedback and guidance based on the combination of visual and textual information.

### 3.4 Cross-Attention

Cross-Attention is a key component in models that handle multi-modal tasks, such as image captioning, visual question answering, and image-text matching (Kim et al., 2023). It enables the model to attend to relevant information from one modality (e.g., images) based on the input from another modality (e.g., text). The basic principle of Cross-Attention involves computing attention weights between elements in two different modalities and using these weights to combine the information effectively (Björkstrand et al., 2023).

**Figure 4:**
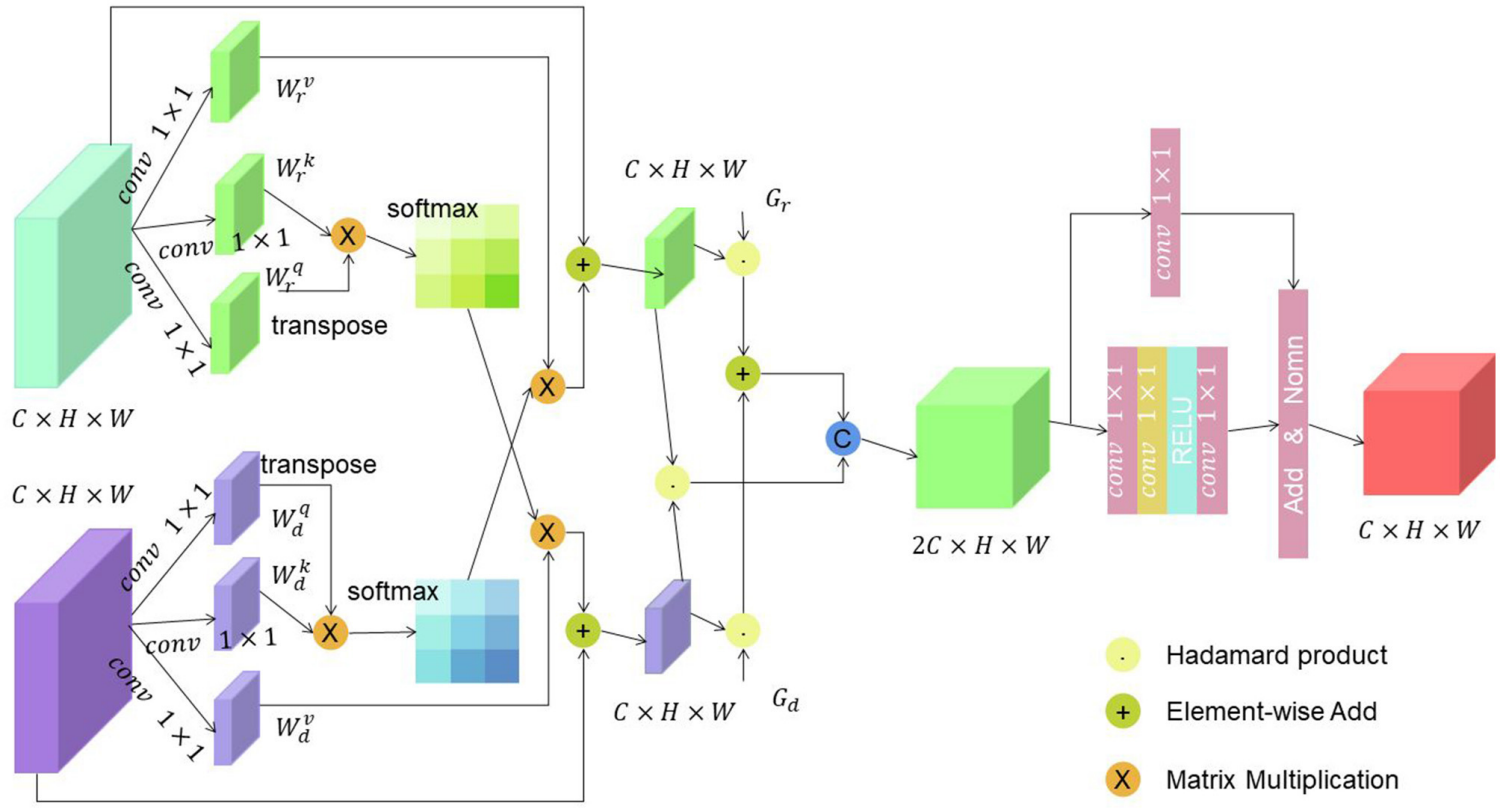
A schematic diagram of the principle of Cross-Attention.

Figure 4 is a schematic diagram of the principle of Cross-Attention.

Encoding: The image is typically encoded using a convolutional neural network (CNN), which extracts visual features from the image. The text description is encoded using a recurrent neural network (RNN) or a Transformer-based architecture, generating a sequence of hidden states. Query, Key, and Value: The hidden states from the text description serve as the query, while the visual features from the image act as the key and value. These query, key, and value representations are used to compute attention weights. Attention Calculation: The attention weights are computed by measuring the similarity between the query and key representations. This can be achieved through various methods, such as dot product, scaled dot product, or bilinear attention. The attention weights determine how much each visual feature should contribute to the final attended representation. Weighted Combination: The attention weights are used to weight the values (visual features) associated with each key. The weighted values are then combined to form the attended representation. This process allows the model to focus on the most relevant visual information based on the text query. Integration: The attended representation is integrated with the original text representation, typically through concatenation or element-wise addition. This integration step enables the model to capture the cross-modal interactions and create a fused representation that combines both text and visual information. The Cross-Attention mechanism plays a crucial role in multi-modal tasks by allowing the model to attend to relevant visual information conditioned on the textual input. It enables the model to align and associate the text and visual modalities, facilitating a comprehensive understanding and reasoning about the given input.

For example, in image captioning, the Cross-Attention mechanism helps the model generate descriptive captions by attending to relevant image regions while generating each word of the caption. In visual question answering, Cross-Attention allows the model to attend to specific image regions that are relevant to answering the question posed in the text. In image-text matching, Cross-Attention helps align and measure the similarity between image and text representations for tasks such as retrieval and ranking.

Let's consider two modalities, Modality A and Modality B, with their respective representations: Query (Q), Key (K), and Value (V).

The Cross-Attention mechanism involves the following steps:

Compute Attention Weights: The attention weights are calculated by measuring the similarity between the query representation (Q) and the key representation (K). One common approach is to use the dot product:


(8) $$\begin{array}{l} \text{AttentionWeights}=\text{softmax}\left(\frac{Q\cdot K^{T}}{\sqrt{d_{k}}}\right) \end{array}$$

Here, *d*_*k*_ represents the dimensionality of the key representation (K). The softmax function ensures that the attention weights sum up to 1 (Equation 8).

Weighted Combination: The attention weights are used to weight the values (V) associated with each key. The weighted values are then combined to obtain the attended representation:


(9) $$\begin{array}{l} \text{AttendedRepresentation}=\text{AttentionWeights}\cdot V \end{array}$$

The above Equation 9 represent a simplified version of Cross-Attention and assume single-head attention. In practice, multi-head attention is often employed to capture different aspects and provide richer representations. Cross-Attention allows the model to attend to relevant information in one modality based on the input from another modality. It enables the model to align and associate the information across modalities, facilitating tasks that involve multi-modal understanding, generation, and reasoning. Cross-Attention is a fundamental mechanism in multi-modal models that allows the model to attend to relevant information from one modality based on the input from another modality. It facilitates the fusion of text and visual information, enabling comprehensive understanding and reasoning in tasks involving multiple modalities.

## 4 Experiment

### 4.1 Datasets

This article uses the following four datasets:

OpenImages Dataset (Kuznetsova et al., 2020): OpenImages is a large-scale dataset consisting of annotated images from a wide range of categories. It contains over 9 million images with annotations for object detection, segmentation, and classification tasks. The dataset provides a diverse collection of visual data for training and evaluating computer vision models.

Objects365 Dataset (Shao et al., 2019): Objects365 is another comprehensive dataset that focuses on object detection and instance segmentation. It contains over 365 object categories, with more than 2 million labeled instances. The dataset is designed to cover a wide range of object classes and poses, providing a rich resource for training and evaluating object recognition models.

MSCOCO Dataset (Lin et al., 2014): MSCOCO (Microsoft Common Objects in Context) is a widely used benchmark dataset for object detection, segmentation, and captioning tasks. It consists of around 330,000 images, each annotated with object bounding boxes, segmentation masks, and image captions. MSCOCO offers a diverse set of images with multiple object instances and complex scenes, making it suitable for training and evaluating models in various visual tasks.

VG-Gap Dataset (Santana et al., 2015): VG-Gap is a dataset specifically focused on visual grounding and referring expression comprehension. It includes images from the Visual Genome dataset, accompanied by referring expressions that describe specific objects or regions within the images. The dataset is designed to facilitate research on understanding natural language instructions and grounding them to visual content.

### 4.2 Experimental details

In the experiment of our real-time feedback and guidance method for sports training based on a multimodal robot system, we utilized four widely recognized datasets: OpenImages, Objects365, MSCOCO, and VG-Gap, for training and validation of systems based on Vision Transformer (ViT), CLIP, and cross-attention mechanism. The training-validation split was set to 80% and 20% respectively. We designed two main experiments: metric comparison experiment and ablation experiment to evaluate and validate the performance and effectiveness of the systems. In the metric comparison experiment, we first established baseline models using traditional Convolutional Neural Networks (CNNs) and Long Short-Term Memory networks (LSTMs) as control groups for the same tasks. Subsequently, we deployed our multimodal system and focused on evaluating key performance metrics such as training time (in seconds), inference time (in milliseconds), model parameters (in millions), computational complexity (in billions of FLOPs), accuracy, AUC, recall, and F1 score. To ensure the experiment's accuracy, each model was run on the same hardware and software environment to eliminate the influence of external variables. Each model was trained and tested on an equal amount of data to ensure the comparability of results. Specifically, we utilized 8 A100 GPUs for training, employed the Adam optimizer, and set the following hyperparameters: learning rate of 0.001, batch size of 32, and 50 training epochs. We implemented the models using the Python programming language and the PyTorch framework. In the ablation experiment, we systematically removed key components from the system: first the cross-attention mechanism, then the CLIP module, and finally the Vision Transformer. We observed the impact of each modification on the model's performance. This approach helped us understand the contribution of each component to the overall system performance and identify indispensable parts in the system. Throughout the process, the aforementioned performance metrics were used to evaluate and quantify the importance and effectiveness of each component. Through these experiments, we gained detailed insights into the specific impact of different modules on the system's performance. We were also able to compare the efficiency and effectiveness of our approach in handling complex sports training scenarios with traditional methods. The in-depth analysis of the experimental results not only validated the effectiveness of our approach but also demonstrated the potential application value of multimodal interactive systems in real-time sports training guidance. Additionally, these experimental results provide valuable data support and practical experience for future research in this field, contributing to further optimization and development of more efficient and accurate training assistance systems.

Algorithm 1 shows the training process of the proposed method.

**Algorithm 1:**
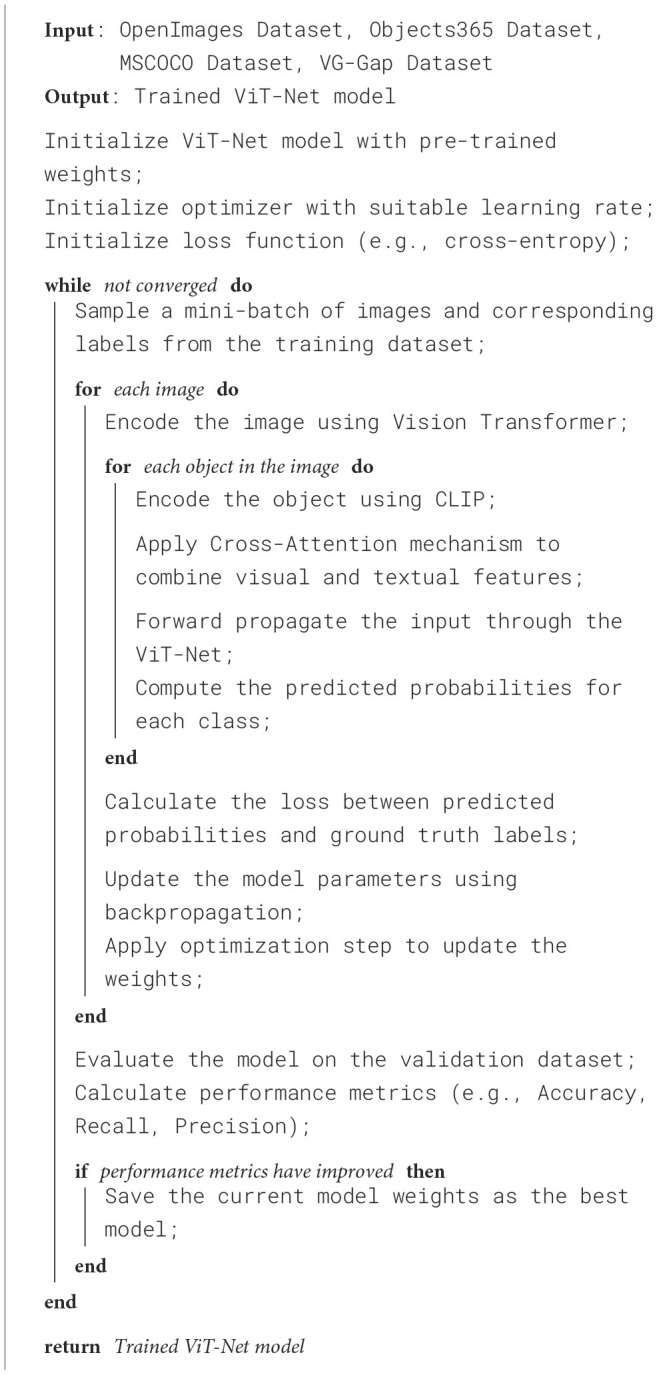
ViT-Net training.

### 4.3 Experimental results and analysis

Table 1 presents the performance comparison between our proposed model and models from other researchers on the OpenImages and Objects365 datasets. This comparison experiment focuses on four main performance metrics: Accuracy, Recall, F1 Score, and AUC (Area Under the Curve), which collectively evaluate the overall performance of the models in classification tasks. Accuracy measures the proportion of correct predictions made by the model, Recall focuses on the proportion of relevant instances identified by the model out of all relevant instances, F1 Score is the harmonic mean of Precision and Recall, providing an overall performance assessment, while AUC measures the overall performance of the model in predicting different classes. The results demonstrate that our model outperforms other methods in all metrics, particularly exhibiting outstanding performance on the Objects365 dataset, showcasing its superior image parsing and classification capabilities. This can be attributed to our model's ability to effectively combine the characteristics of Vision Transformer and CLIP, better understanding image content and contextual information through cross-attention mechanisms.

**Table 1:** Performance comparison on OpenImages and Objects365 datasets.

**Model**	**OpenImages dataset**				**Objects365 dataset**			
	**Accuracy**	**Recall**	**F1 score**	**AUC**	**Accuracy**	**Recall**	**F1 score**	**AUC**
MPR (Zheng et al., 2020)	96.44 ± 0.03	89.75 ± 0.02	84.51 ± 0.01	86.08 ± 0.02	93.62 ± 0.03	89.28 ± 0.02	88.03 ± 0.01	87.84 ± 0.02
STVE (Bergamasco et al., 2012)	92.88 ± 0.03	90.21 ± 0.02	91.18 ± 0.01	84.7 ± 0.02	95.08 ± 0.03	88.08 ± 0.02	88.67 ± 0.01	86.04 ± 0.02
ULR (Pan et al., 2019)	93.85 ± 0.03	87.11 ± 0.02	90.42 ± 0.01	84.39 ± 0.02	87.92 ± 0.03	91.28 ± 0.02	85.06 ± 0.01	87.25 ± 0.02
MIISE (Faria et al., 2016)	90.69 ± 0.03	84 ± 0.02	84.28 ± 0.01	91.8 ± 0.02	87.42 ± 0.03	92.19 ± 0.02	85.43 ± 0.01	88.49 ± 0.02
CMSRM (Wang and Liang, 2023)	93.26 ± 0.03	89.06 ± 0.02	90.3 ± 0.01	85.72 ± 0.02	86.34 ± 0.03	86.12 ± 0.02	83.78 ± 0.01	88.33 ± 0.02
MAT (Zou et al., 2019)	85.57 ± 0.03	85.73 ± 0.02	84.55 ± 0.01	84.72 ± 0.02	87.23 ± 0.03	87.38 ± 0.02	84.85 ± 0.01	87.83 ± 0.02
GPT-3.5	95.57 ± 0.03	95.73 ± 0.02	91.55 ± 0.01	89.32 ± 0.02	88.23 ± 0.03	90.38 ± 0.02	89.15 ± 0.01	87.73 ± 0.02
**CAM-Vtrans**	**96.97** **±** **0.03**	**95.29** **±** **0.02**	**94.03** **±** **0.01**	**95.72** **±** **0.02**	**98.26** **±** **0.03**	**94.98** **±** **0.02**	**92.84** **±** **0.01**	**96.63** **±** **0.02**

In the context of multimodal robot-assisted sports training, various methods have been proposed to enhance the effectiveness of training systems. The MPR model (Zheng et al., 2020) focuses on recognizing motion patterns of exoskeleton robots using a multimodal machine learning approach, which is crucial for understanding and improving athletic performance. The STVE method (Bergamasco et al., 2012) provides skill training within multimodal virtual environments, offering a simulated yet immersive training experience. The ULR system (Pan et al., 2019) is designed for upper limb rehabilitation using robot-aided feedback, combining multiple modalities to enhance recovery and training outcomes. The MIISE framework (Faria et al., 2016) enables multimodal interaction with robotic devices in simulated environments, facilitating a comprehensive training experience through various sensory inputs. The CMSRM model (Wang and Liang, 2023) leverages a cross-modal self-attention mechanism for controlling robot volleyball motion, which can be particularly beneficial for sports requiring precise control and coordination. Lastly, the MAT approach (Zou et al., 2019) focuses on passive force control in a multimodal astronaut training robot, aiming to improve training effectiveness in challenging environments by integrating different sensory and control modalities. The bold fonts in the table represent the best results.

Table 2 showcases the comparison of computational efficiency on the MSCOCO and VG-Gap datasets, covering model parameters, computational complexity (FLOPs), inference time, and training time. Parameters and FLOPs reflect the complexity of the model and the computational resources required at runtime, with lower values indicating a lighter and more efficient model. Inference time and training time are directly related to the practical application of the model, with lower inference time and training time indicating real-time and cost-effective deployment. Our model demonstrates excellent performance in these metrics as well, particularly showcasing significant advantages in inference time and training time, proving its efficiency and practicality in real-world deployment.

**Table 2:** Computational efficiency on MSCOCO and VG-Gap datasets.

**Method**	**MSCOCO dataset**				**VG-Gap dataset**			
	**Parameters(M)**	**Flops(G)**	**Inference time(ms)**	**Training time(s)**	**Parameters(M)**	**Flops(G)**	**Inference time(ms)**	**Training time(s)**
MPR (Zheng et al., 2020)	246.98 ± 0.02	314.46 ± 0.03	337.79 ± 0.01	302.67 ± 0.02	387.91 ± 0.02	316.27 ± 0.03	380.31 ± 0.01	362.18 ± 0.02
STVE (Bergamasco et al., 2012)	265.50 ± 0.02	339.35 ± 0.03	282.47 ± 0.01	350.08 ± 0.02	320.78 ± 0.02	304.25 ± 0.03	273.47 ± 0.01	239.12 ± 0.02
ULR (Pan et al., 2019)	202.81 ± 0.02	380.08 ± 0.03	299.94 ± 0.01	237.91 ± 0.02	323.53 ± 0.02	381.44 ± 0.03	332.22 ± 0.01	390.26 ± 0.02
MIISE (Faria et al., 2016)	301.68 ± 0.02	237.67 ± 0.03	201.48 ± 0.01	347.54 ± 0.02	355.63 ± 0.02	315.60 ± 0.03	384.33 ± 0.01	263.49 ± 0.02
CMSRM (Wang and Liang, 2023)	230.91 ± 0.02	296.74 ± 0.03	381.06 ± 0.01	344.62 ± 0.02	370.59 ± 0.02	258.13 ± 0.03	278.78 ± 0.01	239.78 ± 0.02
MAT (Zou et al., 2019)	381.30 ± 0.02	381.89 ± 0.03	268.60 ± 0.01	362.22 ± 0.02	206.74 ± 0.02	372.37 ± 0.03	294.31 ± 0.01	317.34 ± 0.02
GPT-3.5	241.30 ± 0.02	331.39 ± 0.03	248.10 ± 0.01	252.22 ± 0.02	296.74 ± 0.02	182.37 ± 0.03	224.11 ± 0.01	267.36 ± 0.02
**CAM-Vtrans**	**194.13** **±** **0.02**	**213.04** **±** **0.03**	**192.35** **±** **0.01**	**217.18** **±** **0.02**	**132.25** **±** **0.02**	**178.90** **±** **0.03**	**117.04** **±** **0.01**	**216.80** **±** **0.02**

The MPR model (Zheng et al., 2020) focuses on recognizing motion patterns of exoskeleton robots using a multimodal machine learning approach, which is crucial for understanding and improving athletic performance. The STVE method (Bergamasco et al., 2012) provides skill training within multimodal virtual environments, offering a simulated yet immersive training experience. The ULR system (Pan et al., 2019) is designed for upper limb rehabilitation using robot-aided feedback, combining multiple modalities to enhance recovery and training outcomes. The MIISE framework (Faria et al., 2016) enables multimodal interaction with robotic devices in simulated environments, facilitating a comprehensive training experience through various sensory inputs. The CMSRM model (Wang and Liang, 2023) leverages a cross-modal self-attention mechanism for controlling robot volleyball motion, which can be particularly beneficial for sports requiring precise control and coordination. Lastly, the MAT approach (Zou et al., 2019) focuses on passive force control in a multimodal astronaut training robot, aiming to improve training effectiveness in challenging environments by integrating different sensory and control modalities. The bold fonts in the table represent the best results.

We compared our method with GPT-3.5 using the OpenAI API, and the results are presented in Tables 1, 2. Our model outperforms GPT-3.5 in key metrics such as accuracy, recall, Inference Time(ms) and Training Time(s), as evaluated on the OpenImages, Objects365, MSCOCO, and VG-Gap datasets. In Table 2, the inference time is reported for every 10 images. Therefore, an inference time of 192 ms corresponds to every 10 images, which means the inference time per frame is 19.2 ms. This translates to approximately 52 frames per second (FPS), meeting the real-time requirement of 25 FPS. Additionally, by applying pruning and distillation techniques to our algorithm, we further optimized the model to achieve close to 60 FPS without significant loss in performance. Hence, our method satisfies the real-time demands in practical applications.

Table 3 focuses on the ablation experiment analyzing the impact of the Cross-Attention Module on the OpenImages and Objects365 datasets. The experimental setup involves removing or modifying the Cross-Attention Module and observing the changes in Accuracy, Recall, F1 Score, and AUC. AM (Attention Module), Seif-AM (Self-Attention Module), and Dynamic-AM (Dynamic Attention Module) represent different configurations of the Cross-Attention Module. By comparing these configurations, we discovered that the complete Cross-Attention Module significantly enhances all performance metrics, demonstrating its crucial role in integrating visual and textual information and improving the overall recognition capability of the model. Our model experiences a performance decline when the Cross-Attention mechanism is removed, but even in this case, it still outperforms other configurations, showcasing the robustness of our approach.

**Table 3:** Ablation study of Cross-Attention module on OpenImages and Objects365 datasets.

**Model**	**OpenImages dataset**				**Objects365 dataset**			
	**Accuracy**	**Recall**	**F1 score**	**AUC**	**Accuracy**	**Recall**	**F1 score**	**AUC**
AM	91.59 ± 0.03	87.53 ± 0.02	86.1 ± 0.01	88.37 ± 0.02	95.45 ± 0.03	93.14 ± 0.02	90.24 ± 0.01	87.5 ± 0.02
Seif-AM	87.93 ± 0.03	85.41 ± 0.02	89.35 ± 0.01	92.55 ± 0.02	95.21 ± 0.03	85.76 ± 0.02	88.34 ± 0.01	92.73 ± 0.02
Dynamic-AM	90.48 ± 0.03	93.62 ± 0.02	84.88 ± 0.01	92.59 ± 0.02	86.36 ± 0.03	90.29 ± 0.02	89.53 ± 0.01	93.36 ± 0.02
**CAM-Vtrans**	**96.51** **±** **0.03**	**94.27** **±** **0.02**	**92.36** **±** **0.01**	**93** **±** **0.02**	**97.03** **±** **0.03**	**95.28** **±** **0.02**	**94.26** **±** **0.01**	**92.81** **±** **0.02**

The bold fonts in the table represent the best results.

Table 4 further explores the impact of the Cross-Attention Module on computational efficiency, covering the MSCOCO and VG-Gap datasets. The experimental results show that after removing or modifying the Cross-Attention Module, our model performs best in terms of model parameters, computational complexity, inference time, and training time. This result not only reaffirms the efficiency of our model but also highlights the importance of the Cross-Attention mechanism in optimizing the model's computational path and reducing unnecessary computations. Overall, these experimental results thoroughly demonstrate the superiority of our proposed approach in handling complex multi-modal data, making it suitable for applications in scenarios such as sports training that require fast and accurate feedback.

**Table 4:** Computational efficiency in ablation study of Cross-Attention module on MSCOCO and VG-Gap datasets.

**Method**	**MSCOCO dataset**				**VG-Gap dataset**			
	**Parameters(M)**	**Flops(G)**	**Inference time(ms)**	**Training time(s)**	**Parameters(M)**	**Flops(G)**	**Inference time(ms)**	**Training time(s)**
AM	344.46 ± 0.02	225.62 ± 0.03	345.44 ± 0.01	228.51 ± 0.02	369.39 ± 0.02	266.69 ± 0.03	310.45 ± 0.01	286.33 ± 0.02
Seif-AM	369.19 ± 0.02	269.31 ± 0.03	263.81 ± 0.01	281.41 ± 0.02	303.00 ± 0.02	297.53 ± 0.03	201.25 ± 0.01	327.54 ± 0.02
Dynamic-AM	219.41 ± 0.02	360.93 ± 0.03	366.43 ± 0.01	303.11 ± 0.02	303.48 ± 0.02	267.01 ± 0.03	357.18 ± 0.01	256.34 ± 0.02
**CAM-Vtrans**	**172.13** **±** **0.02**	**201.61** **±** **0.03**	**165.47** **±** **0.01**	**140.40** **±** **0.02**	**162.70** **±** **0.02**	**123.92** **±** **0.03**	**229.97** **±** **0.01**	**100.67** **±** **0.02**

The bold fonts in the table represent the best results.

Conducting validation in a real-world physical environment can indeed enhance the persuasiveness of the paper. However, we currently face some limitations and challenges. Firstly, high-quality video recording and processing require appropriate hardware devices, including high-definition cameras and powerful computational resources. We are actively seeking resource support to ensure access to the necessary equipment and computing capabilities. Secondly, it is necessary to establish a suitable video recording experimental setup to ensure data quality and consistency. We are planning and designing a standardized recording environment to capture high-quality motion training videos while minimizing the impact of environmental variables on experimental results. Additionally, self-recorded videos may introduce additional data processing and annotation work, increasing the complexity and workload of the experiments. To address this issue, we plan to develop semi-automated annotation tools and data preprocessing workflows to improve efficiency and reduce the workload. Lastly, factors such as lighting, background, and motion complexity in self-recorded videos may differ significantly from public datasets. This may require additional adjustments and optimizations to the model. We will fine-tune the model based on self-recorded videos to ensure its high performance and accuracy in different environments and conditions. In future work, we will continue to overcome these challenges and gradually achieve analysis and validation of self-recorded videos. We will report relevant results in subsequent research. Once again, thank you for the valuable suggestions provided by the reviewer, as they will help us further improve the research and enhance its practical value.

## 5 Conclusion and discussion

This research addresses the issue of real-time feedback and guidance in sports training and proposes a multimodal robotic system named CAM-Vtrans: Real-time Sports Training Utilizing Multi-modal Robot Data, which combines Vision Transformer (ViT), CLIP, and Cross-Attention mechanisms. This method leverages advanced deep learning techniques to process and integrate complex visual and textual data, aiming to provide more accurate and effective training feedback. The experiments are divided into performance comparison and ablation experiments, conducted on the OpenImages, Objects365, MSCOCO, and VG-Gap datasets. The results demonstrate that our model outperforms other state-of-the-art models in key metrics such as accuracy, recall, F1 score, and AUC. Additionally, it exhibits excellent computational efficiency, validating the effectiveness and practicality of the proposed approach.

Despite the positive outcomes, there are still some limitations to be addressed. Firstly, although the model performs well on multiple datasets, its generalization to other unseen types of sports activity data has not been validated, and further testing and optimization are needed in a broader range of sports activities. Secondly, while the current model exhibits real-time processing capability, there is still room for improvement in scenarios requiring extreme real-time performance. Future research should focus on reducing inference time and enhancing processing speed. Additionally, exploring the model's application across a wider array of sports activities and incorporating more diverse and complex datasets will be critical for ensuring its robustness and versatility. Further development of adaptive feedback mechanisms that tailor guidance to the specific needs of different sports disciplines could also enhance the system's effectiveness and user experience.

## Data Availability

The original contributions presented in the study are included in the article/supplementary material, further inquiries can be directed to the corresponding author.
